# Supplementary material for: BioConceptVec: Creating and evaluating literature-based biomedical concept embeddings on a large scale
Source: PLoS Comput Biol. 2020 Apr 23;16(4):e1007617. doi: 10.1371/journal.pcbi.1007617 (PMC7237030; doi:10.1371/journal.pcbi.1007617)
Supplement: S1 Table — (DOCX) [file pcbi.1007617.s001.docx]

S1 Table. Evaluation results of the performance of the NER tools in PubTator on the concept types targeted in our study.

| NER tool | Concept Type | Evaluation Dataset | F1 (%) |
| --- | --- | --- | --- |
| tmVar [35] | Mutations | tmVar 2.0 [36] | 93.70 |
| GNormPlus [37] | Genes | BioCreative II GN [38] | 86.70 |
| TaggerOne [39] | Chemicals | BioCreative V CDR [40] | 89.50 |
|  | Diseases | NCBI Disease [41] | 83.70 |
|  | Cell Line | BioCreative VI BioID corpus [42] | 83.10 |
| SR4GN [43] | Species | Linnaeus [44] | 85.42 |
