# Supplementary material for: BioConceptVec: Creating and evaluating literature-based biomedical concept embeddings on a large scale
Source: PLoS Comput Biol. 2020 Apr 23;16(4):e1007617. doi: 10.1371/journal.pcbi.1007617 (PMC7237030; doi:10.1371/journal.pcbi.1007617)
Supplement: S2 Table — (DOCX) [file pcbi.1007617.s002.docx]

S2 Table. Hyperparameters of the Artificial Neural Network (ANN) model for the protein-protein interaction prediction.

|  | **Hyperparameter** | **Value** |
| --- | --- | --- |
| Hidden layer | #Layers | 2 |
|  | Layer units | 2000 and 100 |
|  | Activation function | ReLU |
|  | Dropout rate | 0.2 |
| Output layer | Layer units | 1 |
|  | Activation function | Sigmoid |
| Training | Optimizer | Adam |
|  | Learning rate | 0.005 |
|  | Batch size | 2048 (combined-score)  32 (experimental-700) |
|  | Early stop | 5 |
