# Supplementary material for: BioConceptVec: Creating and evaluating literature-based biomedical concept embeddings on a large scale
Source: PLoS Comput Biol. 2020 Apr 23;16(4):e1007617. doi: 10.1371/journal.pcbi.1007617 (PMC7237030; doi:10.1371/journal.pcbi.1007617)
Supplement: S3 Table — (DOCX) [file pcbi.1007617.s003.docx]

S3 Table. Hyperparameters of the SEN model for the drug-drug interaction prediction.

|  | **Hyperparameter** | **Value** |
| --- | --- | --- |
| Input layer | Max number of tokens | 128 |
| Embedding layer | Embedding dimension | 1024 |
| Dense layer | #Layers | 1 |
|  | Layer units | 500 |
|  | Activation function | ReLU |
|  | Dropout rate | 0.5 |
| Output layer | #Layers | 1 |
|  | Layer units | 5 |
|  | Activation function | Softmax |
| Training | Optimizer | Adam |
|  | Learning rate | 0.001 |
|  | Batch size | 64 |
|  | Early stop | 5 |
