# Supplementary material for: BioConceptVec: Creating and evaluating literature-based biomedical concept embeddings on a large scale
Source: PLoS Comput Biol. 2020 Apr 23;16(4):e1007617. doi: 10.1371/journal.pcbi.1007617 (PMC7237030; doi:10.1371/journal.pcbi.1007617)
Supplement: S4 Table — (DOCX) [file pcbi.1007617.s004.docx]

S4 Table. Classification results of PPI predictions on the STRING database for BioConceptVec (cbow) using different hyperparameters. Combined-scores: PPIs that have combined scores are considered positive cases. Experimental-700: PPIs that have experimental scores over 700 are considered positive cases. Default: BioConceptVec (cbow) trained using the default hyperparameters.

|  | **Combined-score dataset** | | | | **Experimental-700 dataset** | | | |
| --- | --- | --- | --- | --- | --- | --- | --- | --- |
|  | **Precision** | **Recall** | **F1** | **AUC** | **Precision** | **Recall** | **F1** | **AUC** |
| Default | 0.8304 | 0.8025 | 0.8162 | 0.9064 | **0.9476** | 0.7981 | 0.8664 | 0.9525 |
| Window size 5 | 0.8285 | 0.8047 | 0.8164 | 0.9064 | 0.9134 | 0.8333 | 0.8715 | 0.9454 |
| Window size 10 | 0.8280 | 0.8094 | 0.8186 | 0.9079 | 0.9220 | 0.8389 | 0.8785 | 0.9496 |
| Vector dimension 100 | **0.8382** | 0.7941 | 0.8156 | **0.9087** | 0.9108 | 0.8323 | 0.8698 | 0.9438 |
| Vector dimension 300 | 0.8224 | **0.8150** | **0.8187** | 0.9061 | 0.8845 | **0.8868** | **0.8856** | **0.9527** |
| Down sampling 1e4 | 0.8287 | 0.8047 | 0.8165 | 0.9061 | 0.9320 | 0.8217 | 0.8734 | 0.9513 |
| Down sampling 1e5 | 0.8268 | 0.8052 | 0.8158 | 0.9053 | 0.9332 | 0.8209 | 0.8734 | 0.9507 |
| Negative samples 2 | 0.8266 | 0.8047 | 0.8155 | 0.9054 | 0.9304 | 0.8263 | 0.8753 | 0.9508 |
| Negative samples 3 | 0.8308 | 0.8007 | 0.8154 | 0.9061 | 0.9106 | 0.8394 | 0.8736 | 0.9485 |
