# Supplementary material for: BioConceptVec: Creating and evaluating literature-based biomedical concept embeddings on a large scale
Source: PLoS Comput Biol. 2020 Apr 23;16(4):e1007617. doi: 10.1371/journal.pcbi.1007617 (PMC7237030; doi:10.1371/journal.pcbi.1007617)
Supplement: S5 Table — (DOCX) [file pcbi.1007617.s005.docx]

S5 Table. Classification results of DDI classification for BioConceptVec (cbow) using different hyperparameters. SEN: the sentence encoder network. Default: BioConceptVec (cbow) trained using the default hyperparameters.

| **Model** | **F1-score on each relation type** | **Overall performance** |
| --- | --- | --- |

|  | **Int** | **Advice** | **Effect** | **Mechanism** | **P** | **R** | **F** |
| --- | --- | --- | --- | --- | --- | --- | --- |

| SEN + Default | **0.5206** | **0.8423** | **0.8191** | **0.8692** | **0.8167** | **0.8161** | **0.8105** |
| --- | --- | --- | --- | --- | --- | --- | --- |
| SEN + Window size 5 | 0.4701 | 0.8048 | 0.7951 | 0.8758 | 0.7943 | 0.7941 | 0.7903 |
| SEN + Window size 10 | **0.5151** | 0.8178 | 0.8002 | 0.8767 | 0.8066 | 0.8043 | 0.7998 |
| SEN + Vector dimension 100 | 0.4548 | **0.8221** | 0.8025 | 0.8470 | 0.7983 | 0.7884 | 0.7865 |
| SEN + Vector dimension 300 | 0.5040 | 0.8070 | 0.8223 | 0.8696 | 0.8117 | 0.8063 | **0.8022** |
| SEN + Down sampling 1e4 | 0.4719 | 0.8144 | 0.8109 | 0.8520 | 0.8014 | 0.7941 | 0.7911 |
| SEN + Down sampling 1e5 | 0.4366 | 0.8159 | 0.7958 | 0.8608 | 0.7938 | 0.7894 | 0.7851 |
| SEN + Negative samples 2 | 0.4894 | 0.8220 | 0.8112 | 0.8594 | 0.8066 | 0.8020 | 0.7969 |
| SEN + Negative samples 3 | 0.4635 | 0.8102 | 0.8275 | **0.8698** | **0.8184** | **0.8104** | 0.8009 |
